# CloneSifter: enrichment of rare clones from heterogeneous cell populations

**DOI:** 10.1186/s12915-020-00911-3

**Published:** 2020-11-24

**Authors:** David Feldman, FuNien Tsai, Anthony J. Garrity, Ryan O’Rourke, Lisa Brenan, Patricia Ho, Elizabeth Gonzalez, Silvana Konermann, Cory M. Johannessen, Rameen Beroukhim, Pratiti Bandopadhayay, Paul C. Blainey

**Affiliations:** 1 Broad Institute of MIT and Harvard, Cambridge, MA 02142 USA; 2 Department of Physics, MIT, Cambridge, MA 02142 USA; 3 Present address: 10x Genomics, Pleasanton, CA 94588 USA; 4 Present address: Arbor Biotechnologies, Cambridge, MA 02140 USA; 5 Present address: Casma Therapeutics, Cambridge, MA 02139 USA; 6 Dana-Farber/Boston Children’s Cancer and Blood Disorders Center, Boston, MA 02115 USA; 7 Salk Institute, La Jolla, CA 92037 USA; 8 Present address: Novartis Institutes for BioMedical Research, Cambridge, MA 02139 USA; 9 Division of Medical Oncology, Dana-Farber Cancer Institute, Boston, MA 02115 USA; 10 Department of Medicine, Harvard Medical School, Boston, MA 02115 USA; 11 Department of Pediatrics, Harvard Medical School, Boston, MA 02115 USA; 12 Department of Biological Engineering, MIT, Cambridge, MA 02142 USA; 13 Koch Institute for Integrative Cancer Research at MIT, Cambridge, MA 02142 USA

**Keywords:** Cellular heterogeneity, Barcode targeting, Viable clone-specific cells recovery, Clonal fitness tracking, CRISPR sgRNA-barcode DNA library

## Abstract

**Background:**

Many biological processes, such as cancer metastasis, organismal development, and acquisition of resistance to cytotoxic therapy, rely on the emergence of rare sub-clones from a larger population. Understanding how the genetic and epigenetic features of diverse clones affect clonal fitness provides insight into molecular mechanisms underlying selective processes. While large-scale barcoding with NGS readout has facilitated cellular fitness assessment at the population level, this approach does not support characterization of clones prior to selection. Single-cell genomics methods provide high biological resolution, but are challenging to scale across large populations to probe rare clones and are destructive, limiting further functional analysis of important clones.

**Results:**

Here, we develop CloneSifter, a methodology for tracking and enriching rare clones throughout their response to selection. CloneSifter utilizes a CRISPR sgRNA-barcode library that facilitates the isolation of viable cells from specific clones within the barcoded population using a sequence-specific retrieval reporter. We demonstrate that CloneSifter can measure clonal fitness of cancer cell models in vitro and retrieve targeted clones at abundance as low as 1 in 1883 in a heterogeneous cell population.

**Conclusions:**

CloneSifter provides a means to track and access specific and rare clones of interest across dynamic changes in population structure to comprehensively explore the basis of these changes.

**Supplementary information:**

**Supplementary information** accompanies this paper at 10.1186/s12915-020-00911-3.

## Background

The response of a heterogeneous population to selection pressure is shaped by the growth dynamics of individual clones within the population. Rare clones can play a decisive role in the outcome of selection. Examples include evasion of antiretroviral therapy by rare HIV variants [1], expansion of drug-resistant cancer cells under chemotherapy [2], and seeding of metastases by clonal tumor cells [3, 4]. In addition, comparison of such selected clones with low-fitness clones that perished under selection is likely to provide further insight. Studying how genetic and epigenetic differences affect the survival or disappearance of individual clones during selection provides an opportunity to understand both how the selective process operates and how populations are reshaped by selection. In particular, identifying causal drivers of clone fitness could give rich insights into the molecular mechanisms of selection and suggest potential interventions.

Both heritable and plastic cellular features can drive selection outcomes. For example, mutagens such as DNA-damaging chemotherapies can change genetic features, and epigenetic states can rapidly shift in response to drug exposure [5] or environment [6]. Metastatic clones may alter their epigenetic profiles upon seeding a metastatic site [7], obscuring the preexisting plastic features that enabled them to metastasize. However, existing methods to identify these features tend to rely on comparing populations in bulk before and after selection, which limits their usefulness in identifying pre-existing features that changed during selection. Moreover, whereas it is possible to characterize clones that survived these processes, it is much more difficult to characterize (possibly rare) clones that did not, and further compare these to the untreated ancestors of (possibly rare) higher-fitness clones. A useful alternative approach would be to identify clones based upon their response to selective pressure, and then isolate representative untreated cells from each clone for genomic and functional characterization.

Genomically integrated DNA barcodes provide a scalable methodology to track rare clones by measuring relative barcode abundance over time [8–11]. However, relative clone fitness alone cannot elucidate mechanisms of selection. Single-cell technologies can provide genomic profiles of heterogeneous cells within a population. Clone identity can be incorporated into single-cell RNA-seq (scRNA-seq) profiles by capturing transcribed barcodes, linking clonal history and cell fate [12]. However, single-cell genomic profiling is inherently destructive. Both DNA barcoding and single-cell approaches have a limited ability to probe functional differences between clones, whereas retrieval of viable cells from clones would enable a wide range of genomic and functional analyses.

Here, we report CloneSifter, an experimental system that permits tracking, selection, and recovery of arbitrarily chosen, viable clones from a cell population. CloneSifter employs a diverse library of single-guide RNAs (sgRNAs). In the absence of spCas9 activity, these serve as inert barcodes for tracking cells. In the presence of spCas9, these sgRNAs direct spCas9 in a clone-specific fashion to activate a reporter. spCas9-dependent reporter expression permits the physical isolation of specific cells within a population while preserving cell viability. This methodology allows for the enrichment, isolation, and comparative analysis of specific clones at any stage of evolution. Isolated cells can then be characterized by downstream functional assays, such as phenotypic characterization, genetic perturbation, or small molecule screens, thus enabling comprehensive analysis of how pre-existing clonal features affect cells.

## Results

### Overview of the barcoding and retrieval strategy

**Fig. 1:**
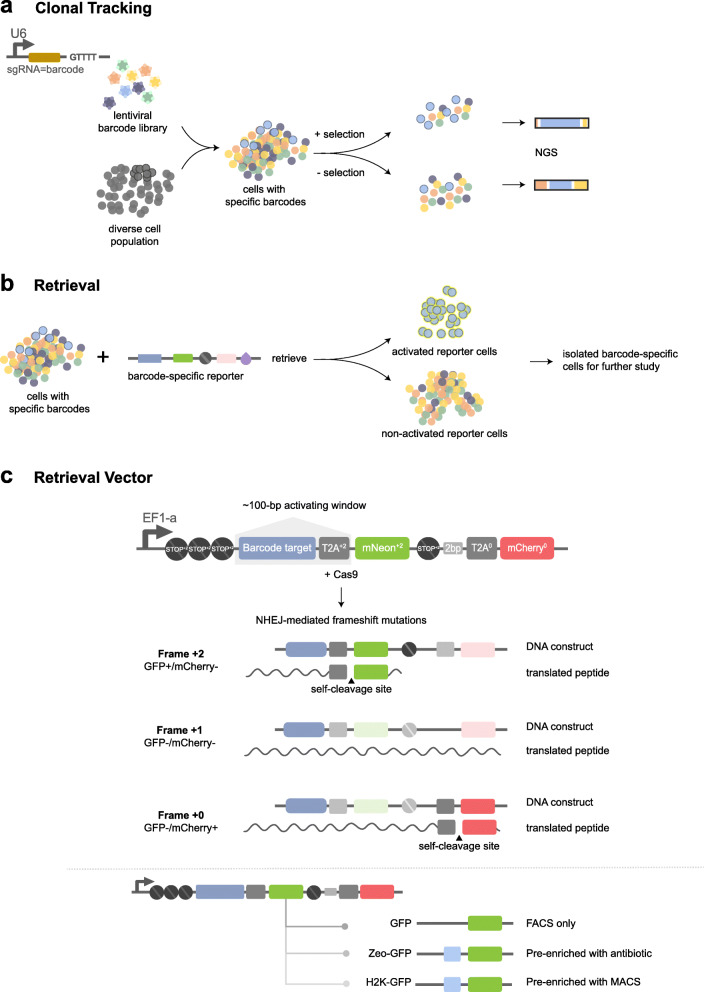
Overview of the strategy for tracking and retrieving the ancestral clones within a heterogeneous population. **a** Tracking clonal response to selection (e.g., ± drug) using a lentiviral sgRNA-barcode library. Clonal fitness profiles can be estimated from barcode enrichment across replicates within each condition. **b** Clones of interest may be retrieved from the ancestral (untreated) population using a retrieval vector containing a targeting region matched to the clone sgRNA-barcode. Nuclease activity at the target region activates a fluorescent marker that can be detected with FACS. **c** Diagram of the frameshift retrieval vector. In cells from the clone of interest, where the sgRNA-barcode and barcode targets are matched, spCas9-mediated cleavage can induce a − 1/+ 2 frameshift, activating reporter expression and inactivating mCherry expression. GFP+/mCherry- cells can be isolated by FACS. Additional reporter genes enable pre-enrichment such as antibiotic selection (e.g., zeocin) or affinity selection (e.g., H2K surface epitope) prior to FACS

To enable tracking and retrieval of clones within a heterogeneous population, we designed a selectable barcode strategy that allows for recovery of viable cells with clone-specific barcodes. In this system, each clone is tagged with a library of random CRISPR sgRNAs [13]. In the absence of spCas9 expression, the sgRNA-barcodes serve as inert labels that are propagated upon cell division, similar to previously reported clonal barcoding strategies [5, 9]. The relative abundance of each clone can be quantified by deep sequencing of the DNA-integrated sgRNA-barcode. The relative fitness of clones can then be determined by sequencing sgRNA-barcodes over time. In line with previous clonal barcoding work, we focus here on populations before and after drug selection. By expanding the ancestral barcoded population and splitting the daughter cells into replicate selection assays, clone-specific fitness differences can be estimated (e.g., clones with a drug-dependent fitness advantage) (Fig. 1a).

We designed this system so that specific clones can be recovered from a barcoded population using a retrieval vector with a target site matching the sgRNA-barcode of interest (Fig. 1b). Introducing spCas9 nuclease leads to double-strand DNA breaks at the target site specifically in the clone that expresses the corresponding sgRNA-barcode. DNA repair generates frameshift mutations at the target site, which may shift the translation frame of one or more downstream reporters [14] (Fig. 1c). Activation of the retrieval reporter can result in both gain and loss of reporter expression (e.g., a shift that brings a GFP reporter into frame and an RFP reporter out of frame).

### An sgRNA-barcode library enables tracking clonal subpopulations

We generated two high complexity sgRNA-barcode libraries using fully degenerate oligonucleotide templates of either 20- or 26- nucleotides (nt). Deep sequencing estimated that the 26-nt plasmid library contained ~ 4.8 million unique barcodes (Additional file 1: Fig. S1a). To test the clone tracking capacity of sgRNA-barcodes, we applied the 26-nt barcode library to monitor clonal resistance to the BET-bromodomain inhibitor JQ1, in D458, a MYC-amplified medulloblastoma cell line known to contain pre-existing resistant clones to BET-bromodomain targeting therapeutic agents (a chemotherapeutic) [5]. We first transduced 4 million D458 cells with the 26-nt barcode library at low MOI (< 0.3) and selected with puromycin. To ensure a high fraction of barcodes corresponding to unique clones, we restricted the population size to ~ 10^5^ barcodes (Method: Barcoding of HeLa and D458).

**Fig. 2:**
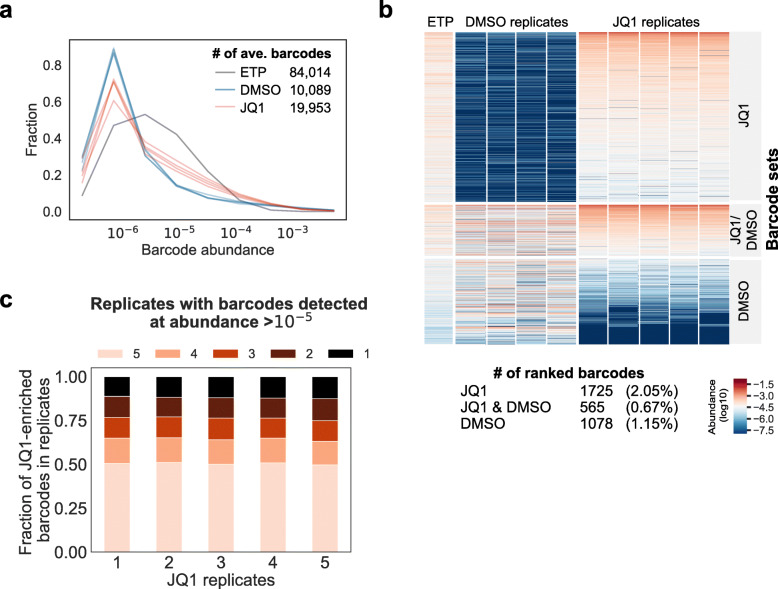
Tracking clonal dynamics in D458 cells using a 26 nt sgRNA-barcode library. **a** Relative barcode abundance in D458 cells before treatment (early time point, ETP) and after treatment with 2 μM JQ1 (5 replicates) or DMSO vehicle (5 replicates). **b**, **c** The sgRNA-barcode library is able to track a heritable phenotype. **b** Comparison of barcode abundance across conditions for barcodes enriched in JQ1, DMSO, or JQ1 and DMSO replicates. Barcode enrichment was defined based on the median rank across replicates (Methods). **c** The majority of JQ1-enriched barcodes were detected across all replicates at an abundance > 10^−5^. The raw barcode read counts are provided as a CSV file in Additional file 5: Table S7-barcode_counts.csv and the raw histograms for barcode counts are available in Supporting Data 2: barcode histograms [15]

We expanded the barcoded D458 population and split it into replicates that were treated with either 2 μM JQ1 or DMSO only (vehicle control). The population of cells treated with JQ1 decreased, then rebounded (Additional file 1: Fig. S1b). After 63 days of either JQ1 selection or DMSO treatment, the replicate populations were harvested and barcode abundance was quantified by NGS. Deep sequencing at the time of the replicate split (early time point, or ETP) detected 84,014 barcodes prior to drug selection (Fig. 2a). After 52 days, we harvested cells and quantified barcode abundance in each replicate (Additional file 2: Table S4). An average of 1725 barcodes were enriched in JQ1-treated replicates, comprising about 2% of the original barcodes. Approximately 50% of the JQ1-selective resistant barcodes were shared by all replicates (Fig. 2b, c); in contrast, fewer than 30% of barcodes were shared across DMSO replicates (Additional file 1: Fig. S1d). Ninety percent of the barcodes (1611/1788) showed significant differential fitness between DMSO and JQ1 on the basis of *t*-distribution test (Additional file 1: Fig. S1e, green) whereas only 0.04% were significant when replicate labels were scrambled (Additional file 1: Fig. S1e, blue). This result suggests that the shared JQ1-selective resistant barcodes were driven by JQ1-selective pressure in particular and not by background drug-independent fitness differences or random effects. Together, we showed both that these barcodes marked clones with predetermined resistance to JQ1 and that our barcode library enables tracking of clones with such heritable phenotypes within a heterogeneous population. Analysis of barcode enrichments showed no significant biases based on barcode GC content or homology to the genome, suggesting that the sgRNA-barcode library can function similarly to other inert barcoding libraries (Additional file 1: Fig. S1f [16]). Although the 26 nt-barcoding library enables tracking complex clonal populations, increasing the length of sgRNA targeting sequences above 20 nt has been shown to reduce spCas9 activity [17]. Therefore, we opted to employ a 20-nt sgRNA barcode library that we have shown is also able to track evolution of populations under pressure from targeted therapies [18].

### Design of a retrieval vector activated by frameshift mutations

To retrieve viable clones, we designed a frameshift reporter that can be specifically activated by an sgRNA-barcode of interest. This approach relies on the generation of insertion or deletion (indel) mutations by spCas9 nuclease in a target region to shift the translation frame of a reporter cassette, similar to vectors used to monitor gene-editing outcomes [8, 19]. An alternative approach would be to use CRISPRa (dCas9-transcriptional activator) to activate marker expression in a barcode-dependent fashion [20–23, 24]. However, we found that a lentiviral transcriptional activation-based reporter lacked specificity, in part due to a high background level of transcription in a fraction of cells subsequent to genomic integration of the reporter (Additional file 1: Fig. S2a and b). Conversely, frameshift reporters have the potential for extremely high specificity due to the low background rate of activating mutations. We opted to deliver the reporter using a lentiviral system capable of effectively transducing a wide range of cell lines. Lentiviral transduction at low MOI followed by antibiotic selection integrates a single reporter copy into most cells, minimizing the potential for a cell to contain multiple reporters in different frameshift states.

We designed a retrieval vector that gains GFP fluorescence in response to a + 2-frameshift mutation that occurs within a narrow targeting window of ~ 100 bp. The vector contains two cassettes respectively in the + 0 and + 2 translation frames: a selection marker (e.g., blasticidin, Blast) and a fluorescent protein (e.g., mCherry) linked by a T2A self-cleaving peptide in the + 0 frame, and a second fluorescent protein (e.g., GFP) in the + 2 frame. The + 0 cassette (mCherry-T2A-Blast) is located downstream of the + 2 cassette in order to aid in selecting for integrants with the correct initial frame via antibiotic selection (blasticidin) or fluorescence-activated cell sorting (FACS) (mCherry) (Fig. 1c). To minimize the likelihood of background activation, we included triple stop codons in all reading frames immediately upstream of the Kozak translation initiation site. All sequences downstream of the translation initiation site were codon-optimized to eliminate start and stop codons that could interfere with reporter performance (Methods: Retrieval reporter construct).

In order to target a specific barcode, the matching target sequence is cloned into the targeting window between the translation start site and the beginning of the GFP coding sequence. Targeting of spCas9 nuclease by the sgRNA-barcode generates indel mutations in the targeting window. If a + 2 indel occurs, the reading frame shifts such that the + 0 cassette is out of frame, while the + 2 cassette is in-frame, giving rise to GFP expression (Fig. 1c). In addition to GFP, a variety of alternative selection elements, such as antibiotic resistance or surface affinity markers, can be used to assist in enriching cells with + 2 frameshifts (Fig. 1c).

### The retrieval vector is specifically activated by target sgRNA-barcodes

**Fig. 3:**
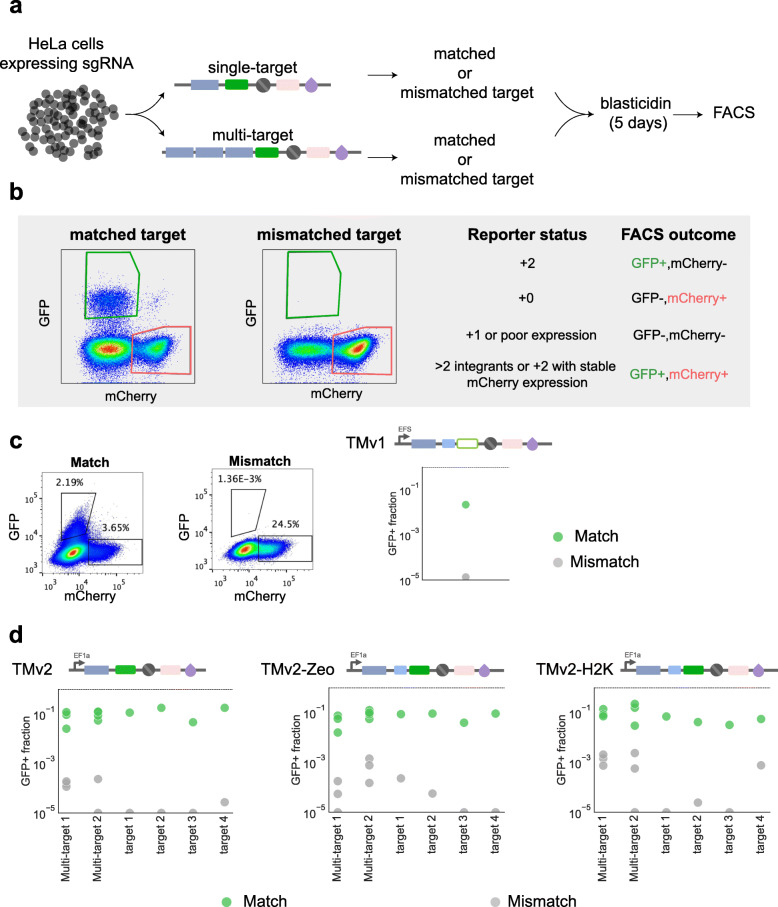
Retrieval vector performance. **a** HeLa cells were transduced with individual sgRNA-barcodes and paired with matched or mismatched barcode targets. **b** Cells with frameshift + 2 and 0 are expected to express GFP and mCherry, respectively, whereas cells with a + 1 frameshift should express neither. **c** Fig. S3 Specificity and sensitivity of the initial retrieval vector design (TMv1). TMv1 demonstrates high specificity, with background activation of around 1–4 in 100,000 cells (matched sequence: GAGACCAGCAGAACCGACAA; mismatched sequence: GCGCAACAGAGAGGGGAGCG). **d** FACS analysis of plots of the TMv2 and TMv2-Zeo retrieval vectors with matched or mismatched barcode targets. Incorporating tandem targets into the retrieval vectors enables multiplexed activation of a single vector by several barcodes. The gating strategy for analysis of the frameshift status of the cells is shown in Additional file 1: Fig. S6. The source data are provided as FCS files in Supporting Data 3: FACS files for Fig. 3 [25]

We generated HeLa-TetR-spCas9 cell lines expressing each individual sgRNA-barcode, so that specificity and sensitivity could be directly assessed by flow cytometry (Fig. 3a). In these experiments, there are 4 possible FACS outcomes, each of which corresponds to a reporter status. For example, GFP+/mCherry- cells are expected to have a + 2-frameshift status (Fig. 3b). A substantial fraction of cells are GFP+/mCherry+, which may indicate multiple reporter integrations with different frameshift statuses, or residual mCherry expression in cells with a + 2 frameshift. To maximize specificity, we consider only GFP+/mCherry- cells as positive reporter activation events. In the same experiment, each retrieval vector was tested with mismatched barcode targets to evaluate specificity (Fig. 3b). We further applied two modifications to improve the activity of our retrieval vector. With the initial version (TMv1), the fraction of GFP+/mCherry- cells was ~ 2% when activating with the matching guide, compared to ~ 0.001% with a mismatched guide control (Fig. 3c). To improve sensitivity, we replaced GFP with mNeonGreen and switched the EFS promoter to a stronger EF1a promoter (TMv2). To allow FACS-independent enrichment, we also expanded the + 0 selection cassette to include either a Zeocin resistance or H2K surface affinity marker upstream of mNeonGreen (TMv2-Zeo, TMv2-H2K). Compared to TMv1, the TMv2 retrieval vector showed approximately 10-fold increased sensitivity at comparable specificity (Fig. 3c, d).

We then systematically evaluated the performance of TMv2 using five randomly selected barcodes from our sgRNA-barcode library and matching targets cloned into TMv2 and TMv2-Zeo. All five barcodes activated mNeonGreen expression from a matching retrieval vector (Fig. 3d). The results showed a low false-positive rate ranging from 0 to 2.7·10^−5^ for TMv2, from 0 to 5.5·10^−4^ for TMv2-Zeo, and from 0 to 2.5·10^−4^ for TMv2-H2K (Fig. 3d). The sensitivity for the matched barcodes ranged from 1.8·10^−1^ to 2.3·10^−2^ for TMv2, from 9.2·10^−2^ to 3.8·10^−2^ for TMv2-Zeo, and from 7.00·10^−2^ to 3.2·10^−2^ for TMv2-H2K, suggesting that the system was capable of high specificity and selectivity (Fig. 3d). For example, TMv2 is projected to be able to enrich clones present as low as 1 in 180,000 (Methods: FACS sample preparation and retrieval vector analysis).

In addition to single barcode reporters, multiplexed activation of several barcodes with one reporter can be achieved by expanding the target sequence to contain targets for multiple sgRNA-barcodes (Fig. 3a–c). To demonstrate multiplexing, we designed retrieval vectors to target three independent sgRNA-barcode sequences (Additional file 1: Table S1). These vectors showed similar sensitivity to those individual sgRNA-barcodes, albeit at 2.6-fold reduced specificity (1.4·10^−3^), possibly due to the increased likelihood of background mutations in the expanded target region (Fig. 3d).

### Identification and viable isolation of rare hygromycin-resistant HeLa cells

We next tested our ability to retrieve drug-resistant and drug-sensitive clones of interest in a well-controlled setting. We engineered hygromycin-resistant HeLa-TetR-spCas9 cells and spiked them into a pool of hygromycin-sensitive HeLa-TetR-spCas9 cells to achieve a final population of cells in which 2% of all cells expressed the hygromycin resistance gene.

**Fig. 4:**
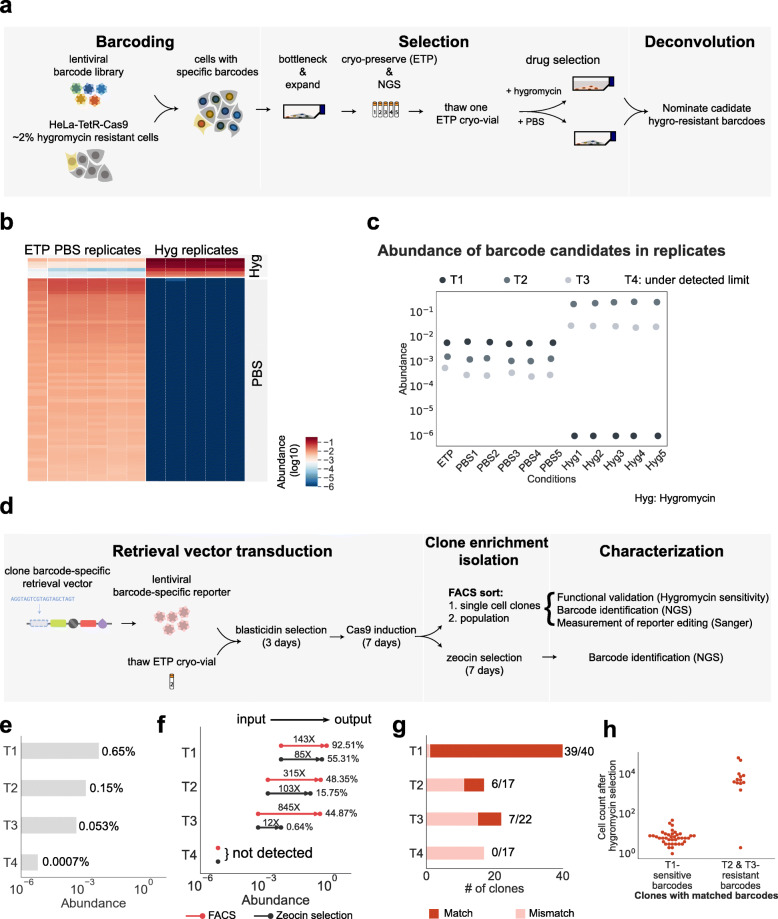
Retrieval of hygromycin-resistant clones from a heterogeneous population of HeLa cells. **a** Workflow to identify resistant clones using a sgRNA-barcode library. (Barcoding) A mixed population of hygromycin-resistant and hygromycin-sensitive HeLa cells was transduced with sgRNA-barcodes. (Selection) The resulting library was bottlenecked to limit barcode complexity, re-expanded, and cryo-preserved to define an early time point (ETP). Cells were then treated with either hygromycin or vehicle control (PBS). Hygromycin-enriched barcodes were determined by NGS. **b** Hygromycin-resistant barcodes were enriched across hygromycin-treated replicates. Barcode abundance for T1 (hygromycin-sensitive barcode candidate), T2 (hygromycin-resistant barcode candidate), and T3 (hygromycin-resistant barcode candidate). The raw barcode read counts are provided as a CSV file in Additional file 5: Table S7-barcode_counts.csv and the raw histograms for barcode counts are available in Supporting Data 2: Barcode histograms [15]. **c** Dot plot showing the abundance of each selected barcode across replicates treated with either PBS or hygromycin. Note that this experiment was done separately from the actual retrieval experiment; T4 is under-detected limited in this original ETP population. **d** Workflow to retrieve resistant clones using the frameshift reporter. (Retrieval vector transduction) Hygromycin-sensitive and resistant candidate barcodes were selected for retrieval, and the matching barcode targets were cloned into the retrieval vector. Cells from the ETP were transduced with barcode-specific retrieval vectors and spCas9 expression was induced. (Clone enrichment and isolation) FACS sorting or zeocin selection was used to enrich for barcodes of interest. Single-cell clones were isolated by FACS. (Characterization) Barcode identification and functional validation. The integrated retrieval vector was sequenced to characterize specific and nonspecific mutations leading to reporter activation. **e** The ETP abundance of each targeted barcode. **f** Population-level enrichment of targeted barcodes using selection by FACS (TMv2) or Zeocin selection (TMv2-Zeo). **g** Fraction of single-cell clones with the targeted barcode. **h** The hygromycin sensitivity of single-cell clones isolated by FACS corresponded to the sensitivity predicted by clonal tracking

We transduced the cells with the 20-nt sgRNA-barcode library at low MOI, and then bottlenecked, expanded, and cryopreserved the cells in replicate vials. Sequencing of one replicate verified the presence of 441 barcodes ranging in abundance from 1 in 100 to 1 in 100,000 (Fig. 4a, Barcoding). To assay for hygromycin resistance, we split the cells and treated them in replicate with either hygromycin or PBS (vehicle) (Fig. 4a, Selection). We then nominated candidate hygromycin-resistant barcodes by comparing the abundance of the barcodes in hygromycin–treated cells to the PBS-treated groups (Fig. 4a, Deconvolution). We found 6 candidate hygromycin-resistant barcodes detected in all 5 hygromycin-treated replicates (Fig. 4b, c, Additional file 3: Table S5).

We carried out retrieval for 4 clonal barcodes: one hygromycin-sensitive barcode candidate (T1) and 3 hygromycin-resistant barcode candidates (T2, T3, and T4) that were represented in the population at frequencies ranging from 1 in 652 (T2) to 1 in 140,000 (T4) (Fig. 4b, d) (Additional file 1: Table S1 and Additional file 3: Table S5). We also analyzed 2 types of control populations: cells transduced with retrieval vectors targeting barcodes not present in the library (T5 and T6, Additional file 1: Table S1), and cells without doxycycline induction of spCas9. Both negative-control groups showed minimal GFP activation upon induction of spCas9 (Additional file 1: Fig. S3). For example, when attempting to retrieve the absent barcode T5 using the TMv2 vector, the GFP+/mCherry- fraction was 1.11·10^−5^ with spCas9 induction and 1.94·10^−5^ without induction (Additional file 1: Fig. S3).

For each sgRNA-barcode, we cloned a matching targeting sequence into the retrieval vectors TMv2, TMv2-Zeo, and TMv2-H2K. To retrieve cells representative of the initial, unselected population, we thawed and expanded barcoded cells preserved at the ETP. Barcoded cells were transduced with TMv2, TMv2-Zeo, or TMv2-H2K and selected with blasticidin for 4 days. Blasticidin was then removed and spCas9 expression was induced with doxycycline for 7 days (Fig. 4d, Retrieval vector transduction).

FACS purification followed by expansion and sequencing greatly enriched T1, T2, and T3. Sequencing of these populations indicated up to 845-fold enrichment of these clones relative to the input fraction, to a minimum purity of 44.87% (T3) and a maximum purity of 92.51% (T1) (Fig. 4e, f, FACS). In addition to the FACS-based enrichment, we also carried out selection using the antibiotic resistance (TMv2-Zeo) and affinity (TMv2-H2K) methods. For TMv2-Zeo, we detected 12–85-fold enrichment (Fig. 4f, Zeocin selection). Clone T4 was not present in the enriched population, suggesting the sensitivity of the retrieval vectors was insufficient to recover viable clones present at frequencies in the population that are smaller than 1 in 140,000 (Fig. 4e, f). TMv2-H2K was excluded from NGS experiments, as it was estimated to have a 1 in 900 false-positive background rate (Additional file 1: Fig. S4). Lastly, we used the multi-target approach with the TMv2-Zeo vector to simultaneously retrieve 4 barcodes (Additional file 1: Table S1, 4-multiplex) or 2 barcodes (Additional file 1: Table S1, 2-multiplex). The 2-multiplex TMv2-Zeo enriched the targeted barcodes by 247-fold and 44-fold (Additional file 1: Table S2). The 4-multiplex TMv2-Zeo enriched 3 out of 4 barcodes, with enrichment rates of 149-fold, 1.9-fold, and 1.4-fold (Additional file 1: Table S2). In both 4- and 2-multiplex targets, we observed a large skew of enrichment toward a single dominant barcode (Additional file 1: Table S2). Given the differences observed among two multi-targets and the two multi-targets characterized by FACS in Fig. 3d (multi-target 1 and multi-target 2), this skew may be reduced by co-optimizing the design of the target sequences, e.g., to balance the rate of activating frameshift mutations from each target.

### Validation of retrieved clones and analysis of sensitivity-limiting background events

In order to confirm that the barcoding and retrieval protocols led to the recovery of clones exhibiting the hygromycin-resistant phenotype, we sorted individual cells transduced with TMv2 into multi-well plates and expanded them as clonal populations (Fig. 4d, Clone enrichment and isolation). We analyzed a total of 132 single-cell clones by deep sequencing their sgRNA-barcodes. We detected two populations: clones with exact matches to the targeted barcodes (52/132 clones) and extensively mismatched clones (barcode edit distance > 9; 80/132 clones) (Fig. 4g and Additional file 1: Fig. S5a, red square). The hygromycin sensitivity of the single-cell clones reflected our expectations based on their barcodes, with the exception of one single-cell clone with a candidate hygromycin resistance barcode (T3) that was sensitive to hygromycin (Fig. 4h and Additional file 1: Fig. S5a, blue square).

To investigate activation of the retrieval vector, we performed Sanger sequencing of 75 clones over a 2-kb region encompassing the translation start site, barcode-specific targeting region, and mNeonGreen coding sequence. As expected, clones with the correct sgRNA-barcode contained + 2 frameshift mutations in the targeting region, with a distribution of indel sizes largely below 10 nt (Additional file 1: Fig. S5b), consistent with repair by non-homologous end-joining following spCas9 cleavage [26]. In agreement with a previous study [27] characterizing large deletions after spCas9 cleavage, we also see a small fraction of larger deletions, for example, a 400-nt deletion in T1 and a 200-nt deletion in T3 (Additional file 1: Fig. S5b). Additionally, we observed a stereotyped ~ 80-nt stereotyped deletion in 21/43 false-positive clones. The deletion is immediately upstream of the mNeonGreen coding sequence (Additional file 1: Fig. S5a, orange square, b and c). Given the presence of this deletion in clones lacking homology between the sgRNA (confirmed by targeted sequencing) and the TMv2 vector, we suspect the deletion results from an spCas9-independent mechanism. We also analyzed 53 clones from a non-targeting control, T5, which also showed a similar type of T2A deletion (Additional file 1: Fig. S5b, T5). Deletions due to lentiviral intra-molecular recombination between homologous regions are well-characterized [28]. However, our codon-optimized retrieval vector lacks substantial homology near the deleted region (no repeated kmers with length > 7), suggesting an alternative mechanism. The false-positive events observed were largely due to the stereotyped deletion. Sorting error likely did not contribute to these false-positive events, as re-analysis of expanded clones showed that all clones contained GFP+/mCherry- cells (Additional file 1: Fig. S5a, green square).

Together, these results indicate that CloneSifter is capable of tracking hygromycin-resistant phenotypes under treatment and enriching rare clones up to 800-fold.

### Retrieval of targeted clones from D458 medulloblastoma cell line

In order to demonstrate the use of CloneSifter in a cancer cell line setting, we turned to D458, a cell line derived from medulloblastoma that is utilized as a model of resistance to the small-molecule chemotherapeutic JQ1 [5]. Moreover, since D458 is a model of group 3 medulloblastoma, which is passaged as 3D neurospheres, we reasoned that this would challenge the limits of CloneSifter in a heterogeneous, patient-derived cancer cell line. We opted to deliver sgRNA-barcodes with the CROPseq vector [29] that enables simultaneous expression of functional sgRNAs and facile detection of sgRNA sequences via single-cell readouts such as single-cell and in situ sequencing [29, 30]. After transduction and selection, we restricted the cell library to various sizes ranging from 1·10^3^ to 3·10^5^. Sequencing showed that the 1·10^4^-barcode library captured the most singly barcoded clonal lineages, as the duplication rate between two independent libraries was 1% (Additional file 1: Fig. S6c). After selecting the barcoded-cells with DMSO, we found 46 barcodes enriched in the DMSO replications, likely due to their increased fitness within the population (Additional file 4: Table S6). We selected one of the DMSO barcodes to retrieve. The initial abundance of the DMSO clone was 1 in 346. To increase the enrichment efficiency, we employed a strategy where cryopreserved cells were recovered in 96 subpools. Sequencing each subpool revealed the optimal subpool to use for subsequent retrieval (abundance of the DMSO barcode increased to 1 in 133) (Additional file 1, Fig. S6d, Subpool sequencing and Retrieval vector transduction, and Additional file 1, Fig. S6e and f).

In order to decrease the background rate of TMv2, two modified versions of TMv2 were tested: exchanging mNeonGreen with mCherry for reporting + 2-frameshift-targeted clones (TMv3), and deleting the T2A cassette in TMv2 (TMv2-deltaT2A). TMv3 did not show improvement in the background rate compared to TMv2 (Additional file 1, Fig. S6a). TMv2-deltaT2A decreased the background rate by 5-fold; however, the sensitivity decreased by 10-fold (Additional file 1, Fig. S6b). We therefore decided to keep the T2A element and moved forward with TMv2 for retrieval experiments. To test the capability of CloneSifter to enrich target clones from the barcoded D458 cell line, we delivered a TMv2 reporter followed by selection with blasticidin for 5 days (Additional file 1, Fig. S6d, Subpool sequencing and Retrieval vector transduction). We then transduced the population with spCas9-BFP virus (MOI ~ 0.42) and cultured cells for an additional 8 days prior to FACS sorting of BFP+/GFP+/mCherry- cells (Additional file 1, Fig. S6d, Retrieval and Barcode validation with NGS). As the CROPseq sgRNA-barcoding system generates mRNA carrying the sgRNA-barcode, we can directly read out sgRNA-barcodes from reverse-transcribed cDNA without isolating gDNA [29]. Directly lysing the sorted single cells for single-cell RNA-Seq followed by amplifying the barcode-targeted region allowed us to immediately validate the barcode sequence in the retrieved single-cell clones. We saw the targeted DMSO clones were greatly enriched, with over 85% purity among retrieved single-cell clones (77/90; Additional file 1: Fig. S6f, blue). This experiment demonstrated the ability to enrich rare lineages from a bona fide cell culture model of clonal heterogeneity in cancer.

## Discussion

We engineered a molecular tool that couples an sgRNA-barcode library for tracking clones with an spCas9-based frameshift reporter to isolate viable cells representing target clones from the population. CloneSifter is one representative of an emerging new class of tools for studying the mechanisms underlying clonal evolution that overcome limitations of bulk methods in resolving the characteristics of rare clones and destructive nature of existing single-cell genomics methods. We showed that our system can accurately track clonal fitness under drug selection and allows efficient retrieval of a targeted set of viable clones at frequencies as low as 1 in 1883. Compared to other systems [21], our CRISPR sgRNA-barcode approach scales to large populations, and we demonstrated that our library is capable of barcoding > 10^5^ clones, while a frameshift retrieval reporter activated by barcode-specific spCas9-mediated mutations enables fluorescence-based enrichment and isolation of clones. The sgRNA-barcode design is especially conducive to multiplexing for simultaneous retrieval of a handful of clones at a time (as shown in Fig. 3c), because it allows straightforward expansion of the activating window to accommodate multiple sgRNA-barcodes.

Isolating clonally barcoded cells from an untreated, ancestral population enables direct testing of mechanisms underlying differential clone fitness. Unlike bulk methods that rely on strong positive selection to enrich cells of interest, our method allows retrieval from clones with any fitness profile, such as slow-growing, persistent, or negatively selected clones. The ability to expand pure populations of target clones enables the use of a broad range of functional and molecular profiling assays. For example, access to pure populations enables high-input assays to determine how epigenetic alterations, such as changes in DNA methylation and chromatin state, affect fitness differences between genetically similar clones. Deep characterization of purified resistant clones is useful in identifying resistant drivers, and through perturbational approaches, the association between these putative drivers and phenotype can be defined. The sgRNA-barcodes can also be readily adapted to existing high-throughput single-cell readouts developed for CRISPR screens, such as single-cell gene expression [29, 31] and optical screening [30].

An important caveat for this and other lentiviral-based DNA barcoding strategies is the possibility of unintended side effects from semi-random lentiviral integration on barcoded clones. Lentiviral integration can either disrupt or increase gene activity, leading to clone-specific effects. Our approach, in which clones of interest are isolated, simplifies the sequencing of the DNA barcode insertion site, which can help rule out integration-driven effects. While introducing the retrieval vector requires an additional lentiviral integration event and manipulation in culture, multiple independent sub-clones can be retrieved per clone of interest, serving as biological replicates for the retrieval process. A second potential confounding factor is sgRNA-barcode sequence-specific effects on clone fitness in the absence of spCas9. However, we found no significant sgRNA homology to the genome or correlation between enrichment in a clone tracking experiment (Additional file 1: Fig. S1f [16]).

Background activation of the frameshift retrieval reporter may hinder applications where the clones of interest exist at low frequency. In principle, a reporter activated by an indel mutation in a 100-nt activating window could have a background rate as low as ~ 1 in 1 billion per cell division, as the rate of naturally occurring indel mutations in human cells is estimated to be ~ 1 in 10^11^ indel/bp/cell division per generation [32–34]. While we found performance was limited by more frequent mutational errors, the high theoretical limit encourages our effort to continue improving our implementation of this approach. We identified stereotyped deletions in the T2A linker region as the primary source of error rate. While sgRNA/spCas9 can introduce large deletions or insertion (up to kilobase) [26], we observed the T2A deletion in clones with non-targeting sgRNAs, suggesting alternative mechanisms. Optimizing the T2A sequence might significantly suppress this particular background source. Alternatively, negative selection against the GFP-containing frame could be applied prior to editing to remove cells with a premature frameshift [35]. To improve sensitivity to both + 1 and + 2 frameshifts, a second reporter cassette could be added in the + 1 frame. Selecting sgRNA-barcodes based on their predicted indel distribution could further increase activation efficiency [36]. Using population-level enrichment methods, such as zeocin selection or FACS-based sorting, we could enrich targeted barcodes to a reasonable purity (~ 50%, Fig. 4f; 85%, Additional file 1: Fig. S5f), a level of enrichment sufficient for many purposes including bulk and single cell analyses. Such enriched populations with identifiable barcodes are ideal samples for single-cell analysis and allow efficient utilization of advanced approaches with limited cellular throughput [37]. Sorting and expanding single-cell clones can increase purity for bulk analyses that require it. However, expanding single cells can be challenging in some cell lines. For example, we observed that a minimum population size of 200 cells was required for D458 cells to maintain normal doubling time. The compatibility of our barcode retrieval vector with single-cell assays (e.g., single-cell RNA-seq) would allow enrichment and characterization of barcoded clones with or without expansion of individually sorted cells.

The CloneSifter retrieval process consists of a series of transduction and sorting processes, which may restrict application in cell types that are sensitive to transduction or are negatively impacted by flow sorting, such as microglia and neurons. However, multiple retrieval formats are enabled by the frameshift reporter system, providing the opportunity to customize the approach for a particular sample type. For example, transduction could be replaced with a non-lentiviral alternative delivery method or surface epitope affinity pull-down could be substituted for flow sorting.

## Conclusions

Clone tracking and retrieval enable deep, mechanistic studies in a wide range of selection scenarios. For example, tracking cells during reprogramming or differentiation protocols would enable isolation and epigenetic characterization of ancestral clones that are predisposed to successful outcomes [12, 38]. Similarly, retrieving untreated cells from clones surviving mutagenic chemotherapy, such as alkylating agents, could address outstanding questions about whether resistance is pre-existing or acquired [39]. Clones can also be targeted based on fitness profiles derived from multiple parallel selection conditions. Altogether, the capability for live clone retrieval enables barcode tracking experiments to advance from observations of clone frequency statistics toward experimentally driven mechanistic studies by providing access to key samples supporting a wide range of genomic and functional assays.

## Methods

### Library construction

Degenerate oligos for sgRNA-barcode library construction were synthesized by IDT and cloned into LentiGuide-Puro [40] by Gibson assembly as previously reported [41] . Approximately 300 μg of Gibson product was transformed into 25 μL of Endura electrocompetent cells (Lucigen). After a 1 h recovery period, 0.1% of transformed bacteria were plated in a 10-fold dilution series on ampicillin plates to determine the number of successful transformants. The remainder of the transformed bacteria were cultured in 50 mL of LB with 50 μg/mL ampicillin for 16 h at 30 °C. Plasmid libraries were extracted using Plasmid MidiPlus kit (Qiagen) and sequenced to a depth of 95 million reads on Illumina Nextseq, corresponding to 13X coverage of 3.9 million barcodes. Lentivirus was prepared as previously reported [41] by transfecting a total of 10 million HEK 293FT cells. The library virus was determined by transduction and puromycin selection in HeLa-Tet-spCas9 cells to contain 600 million infective particles, corresponding to a 153X coverage of barcodes.

### Engineering of a mixed HeLa population with hygromycin-resistant and hygromycin-sensitive cells

HeLa-TetR-spCas9 cells were transduced with a lentiviral ORF construct (pLX_TRC317_PGK-Hygro) containing a hygromycin resistance cassette. After selection with hygromycin (300 μg/ml) for 1 week, hygromycin-resistant cells were spiked into uninfected cells at a ratio of 1:50.

### Barcoding of HeLa and D458 cell lines

HeLa-Tet-spCas9 cells were cultured in DMEM medium supplemented with 10% tetracycline-screened FBS (Hyclone) and 1% penicillin-streptomycin. sgRNA-barcodes were transduced as previously described [41] and selected with 1 μg/mL puromycin for 3 days. The lentiviral multiplicity of infection (MOI) was determined to be between 0.05 and 0.3 for all libraries, so that a majority of cells carry a single integrated sgRNA-barcode. Barcoded cell lines were expanded to a total of 1.0·10^7^ cells and cryopreserved in aliquots of 1.0·10^6^ cells for subsequent drug selection and retrieval.

D458 medulloblastoma cells were cultured in DMEM/F12 media supplemented with 10% FCS and 1% GPS (glutamate, pen-strep). Four million cells were transduced with the sgRNA barcode library (10 wells of 3.0·10^6^ cells with 50ul of virus) by spin infection (1000 g, 120 min, 30 °C). Selection with 1 μg/mL puromycin was initiated 48 h post-transduction and maintained for a total of 3 days.

### Drug resistance experiments: D458 and JQ1

Barcoded D458 medulloblastoma cells (fingerprint verified) were treated with 2 μM JQ1 or DMSO vehicle control in multiple replicate plates (5 x DMSO and 5 x JQ1). Four million barcoded D458 cells were plated in each replicate plate in the presence of DMSO or JQ1. Barcoded D458 cells were also frozen in 10% DMSO/FCS for future retrieval. In addition, cells were collected for DNA-extraction to determine barcode representation at the early time point (ETP). The cells were retreated with compound every 3–4 days. Cells were counted and passaged every 3–4 days, maintaining a minimum representation of 4 million cells. Cells were cultured in DMSO or JQ1 for a total of 52 days prior to harvesting for DNA extraction for barcode sequencing and deconvolution.

### Drug resistance experiments: HeLa and hygromycin

Cells were transduced with the CloneSifter library at MOI < 0.3. Following selection with puromycin, a fixed number of cells were plated (to limit the total number of barcoded clones present). After expansion, cells were frozen in liquid nitrogen (early time point, ETP) in replicates of 1·10^7^ cells. One replicate was thawed for barcoding experiments. Replicate cells were cultured in hygromycin (300 μg/mL) or PBS for 16 days (5 replicates each), after which DNA was extracted from both the ETP control, PBS- and hygromycin-treated replicates for barcode sequencing and deconvolution. At each passage, we ensured the number of cells plated was at least 10-fold the library complexity in order to maintain representation.

### Library deconvolution

Genomic DNA was extracted and prepared for deep sequencing as reported [41]. Libraries were sequenced to a minimum depth of 18 million reads, corresponding to a barcode coverage of > 80X. Counts of sgRNA-barcodes were obtained by filtering for reads containing exact matches to the flanking sequences, and matches with < 3 reads were discarded.

### Clonal fitness measurements

Relative clone abundances were calculated from normalized read counts and clones were ranked by the abundance within each replicate. For the D458 clonal tracking experiment in Fig. 2, we ranked barcodes by the median within-replicate rank and set a cutoff of 2500 to define the barcode sets. We found that the majority of shared-JQ1 barcodes fell into this category (Additional file 1: Fig. S1c). The shared-JQ barcodes were defined as barcodes with an abundance greater than 1 in 100,000 across all JQ1-treated replicates and median abundance in DMSO-treated replicates smaller than 1 in 100,000. NGS data analysis was run with Python 2.7 using libraries numpy 1.13.1, matplotlib 2.1.2, seaborn 0.9.9, and jupyter 4.3.0.

### Retrieval reporter construct

The mNeonGreen, T2A, zeocin resistance, H2K, and blasticidin resistance coding sequences were codon-optimized with silent nucleotide substitutions to remove out-of-frame start and stop codons. Oligos containing targeting barcode sequences and PAM (NGG) matching barcodes of interest were synthesized (IDT) and cloned into frameshift reporter plasmids by golden gate assembly. All targeting barcode sequences were filtered to have < 70% GC content, no more than 4 consecutive repeated bases. Sequences were placed in sense or antisense orientation as required to avoid introducing stop codons in the + 0 or + 2 translation frames. Lentivirus was prepared as previously described [41] and transduced into barcoded HeLa-Tet-spCas9 cells at an MOI of < 0.3. After 4 days of selection with 10 μg/mL blasticidin, 1 μg/mL doxycycline was added to induce spCas9 expression. Cells were harvested for deep sequencing as previously reported [41].

### FACS sample preparation and retrieval vector analysis

HeLa cells were carefully washed with PBS and trypsinized with TrypLE Express (Gibco) for 5 min. DMEM media contained 10% FBS and 1% Penicillin/Streptomycin was used to neutralize trypsin prior to FACS analysis. Fluorescent protein expression was measured on a Cytoflex flow cytometer. FlowJo V10 was used for analysis. Populations were sorted with high-purity mode on a SONY-SH800 FACS machine and expanded for 2 weeks before deep sequencing. All analyzed populations were first gated on FSC-A/FSC-H and FSC-A/SSC-A to identify singlets and cells respectively (Additional file 1: Fig. S7). Projected enrichment efficiency of the retrieval vector (ratio of final abundance *X*_1_ to initial abundance *X*_0_) was defined based on the experimentally measured sensitivity (fraction of GFP+/mCherry- events in a population containing only the target sgRNA-barcode) and specificity (1 - fraction of GFP+/mCherry- events in a population containing only mismatched sgRNA-barcodes) as follows: *X*_1_/*X*_0_ = sensitivity/(*X*_0_ × sensitivity + (1 − *X*_0_) × (1 − specificity)). For small values of *X*_0_, corresponding to barcodes with low initial abundance, the projected enrichment efficiency is approximately: sensitivity/(1 − specificity).

### Characterization of clones

FACS-sorted clones were trypsinized in place 3 days after sorting and further expanded for ~ 7 days. GFP and mCherry expression for each clone was measured by a flow cytometer equipped with a multi-well plate sampler (Beckman Coulter CytoFLEX). To determine hygromycin sensitivity, the clones were treated with or without 300 μg/ml hygromycin and the media was replenished with fresh hygromycin every 3 days for 7 days. Cell counts were obtained by flow cytometry. For Sanger analysis, a 2-kb region of the lentiviral transgene was PCR-amplified from the EF1a promoter (forward strand, primer pTM_negative_fwd) and from the Blast gene (reverse strand, primer pTM_negative_rev) and sequenced with primer pTM_sanger_primer (Additional file 1: Table S3).

### Analysis of frameshift status and indel calculation

For each clone, we used the corresponding unedited retrieval vector as a reference sequence for alignment of Sanger sequencing traces. We determined the location of insertion/deletion/substitution mutations by manual inspection and summarized the mutation as follows. The mutation length (d) was calculated as the difference between the length of the Sanger sequenced vector and the reference sequence, restricted to a window defined by high Sanger quality. The frameshift status was defined as (d) modulo 3. To identify the indel location and length, we focused on the region between the translational start site and the mNeonGreen coding sequence. We then identified the first (reporter.prefix) and last (reporter.suffix) bases of the prefix and suffix sequences of the edited retrieval vector and the first (reference.prefix) and last (reference.suffix) bases of the prefix and suffix sequences of the corresponding region of the reference locus. We then defined the “query gap” and “reference gap” as the difference between the prefix and suffix bases of the edited retrieval vector and the reference locus, respectively (query gap = reporter.suffix − reporter.prefix; reference gap = reference.suffix − reference.prefix). The overall indel outcome was considered an insertion if the query gap exceeded the reference gap; otherwise, it was considered a deletion.

### Cell line authentication

HeLa-TetR-spCas9 cells were a gift from Iain Cheeseman (MIT, Whitehead Institute). D458 cell-lines were a gift from Dr. Bigner (Duke University). To ensure the authenticity of cell lines, we performed Fluidigm SNP-based fingerprinting of each model cell line prior to screening. Cells were routinely tested to exclude the presence of mycoplasma.

## Supplementary Information

**Additional file 1:** **Fig. S1.** sgRNA-barcode library representation, GC bias, and human genome off-targets. **Fig. S2.** Transcriptional activation-based retrieval reporter. **Fig. S3.** Negative-control groups showed minimal GFP+/mCherry- fraction. **Fig. S4.** Performance of TMv2-H2K performance in a pooled population. **Fig. S5.** Characterization of retrieved clones. **Fig. S6.** Retrieval vector optimization and retrieval of targeted clones from D458 cells. **Fig. S7.** Gating strategy for analysis of cells with activated frameshift reporter. **Table S1.** Table of the clonal barcode candidates, multi-target barcodes and non-targeting barcodes in Fig. 3d, Fig. 4 and Fig. S3. **Table S2.** Enrichment levels of barcodes that are retrieved with a 4-multiplex TMv2-Zeo or a 2-multiplex TMv2-Zeo. **Table S3.** Table of primer sequences used for amplifying 2 kb-lentiviral transgene and for Sanger sequencing.

**Additional file 2:** **Table S4.** Table of sgRNA-barcode sequences from the D458 clonal tracking experiment. The abundance of each sgRNA-barcode was calculated from normalized read counts and transformed by a base-10 logarithm (Methods: Clonal fitness measurements). Median rank and median values of each sgRNA-barcode in each condition across replicates are listed. Identified barcode sets including JQ1, DMSO and JQ1 & DMSO are listed in the last column.

**Additional file 3:** **Table S5.** Table of sgRNA-barcode sequences from the HeLa clonal tracking experiment. The abundance of each sgRNA-barcode was calculated with normalized read counts and transformed by a base-10 logarithm (Methods: Clonal fitness measurements). Median ranks and median values of each sgRNA-barcode in each condition across replicates are listed. Identified barcode sets including Hygromycin and PBS are listed in the last column.

**Additional file 4:** **Table S6.** Table of sgRNA-barcode sequences from the D458 clonal tracking experiment with CROP-seq based sgRNA-barcode library. The abundance of each sgRNA-barcode was calculated with normalized read counts and transformed by a base-10 logarithm (Methods: Clonal fitness measurements). Median ranks and median values of each sgRNA-barcode in each condition across replicates are listed. Identified barcode sets including JQ1 and DMSO are listed in the last column.

**Additional file 5:** **Table S7.** Barcode_counts.

## Data Availability

The retrieval vectors (pLenti_TMv2 #131761 and pLenti_TMv2-Zeo #131762) are deposited at Addgene. The barcode read counts table for Fig. 2, Fig. 4, and Fig. S6 are available in Additional file 5 – Table S7-barcode_counts.csv. Python scripts used for NGS analysis are available in Supporting Data 1 in the figshare repository, 10.6084/m9.figshare.12932729 [42]. The raw histograms for barcode counts are available in Supporting Data 2 in the figshare repository, 10.6084/m9.figshare.12932791 [15]. FCS files containing flow cytometry data supporting the conclusions of Fig. 3 are available in Supporting data 3 in the figshare repository, 10.6084/m9.figshare.12895061 [25]. Sanger sequencing datasets supporting the conclusions of Fig. S5 are available in Supporting data 4 in the figshare repository, 10.6084/m9.figshare.12932794 [43]. Raw sequencing data is available in Supporting data 5 in the figshare repository, 10.6084/m9.figshare.12932546 [44].
