# Supplementary material for: CloneSifter: enrichment of rare clones from heterogeneous cell populations
Source: BMC Biol. 2020 Nov 24;18:177. doi: 10.1186/s12915-020-00911-3 (PMC7687773; doi:10.1186/s12915-020-00911-3)
Supplement: Supplementary file 1 — Additional file 1: Fig. S1. sgRNA-barcode library representation, GC bias, and human genome off-targets. Fig. S2. Transcriptional activation-based retrieval reporter. Fig. S3. Negative-control groups showed minimal GFP+/mCherry- fraction. Fig. S4. Performance of TMv2-H2K performance in a pooled population. Fig. S5. Characterization of retrieved clones. Fig. S6. Retrieval vector optimization and retrieval of targeted clones from D458 cells. Fig. S7. Gating strategy for analysis of cells with activated frameshift reporter. Table S1. Table of the clonal barcode candidates, multi-target barcodes and non-targeting barcodes in Fig. 3d, Fig. 4 and Fig. S3. Table S2. Enrichment levels of barcodes that are retrieved with a 4-multiplex TMv2-Zeo or a 2-multiplex TMv2-Zeo. Table S3. Table of primer sequences used for amplifying 2 kb-lentiviral transgene and for Sanger sequencing. [file 12915_2020_911_MOESM1_ESM.docx]

**
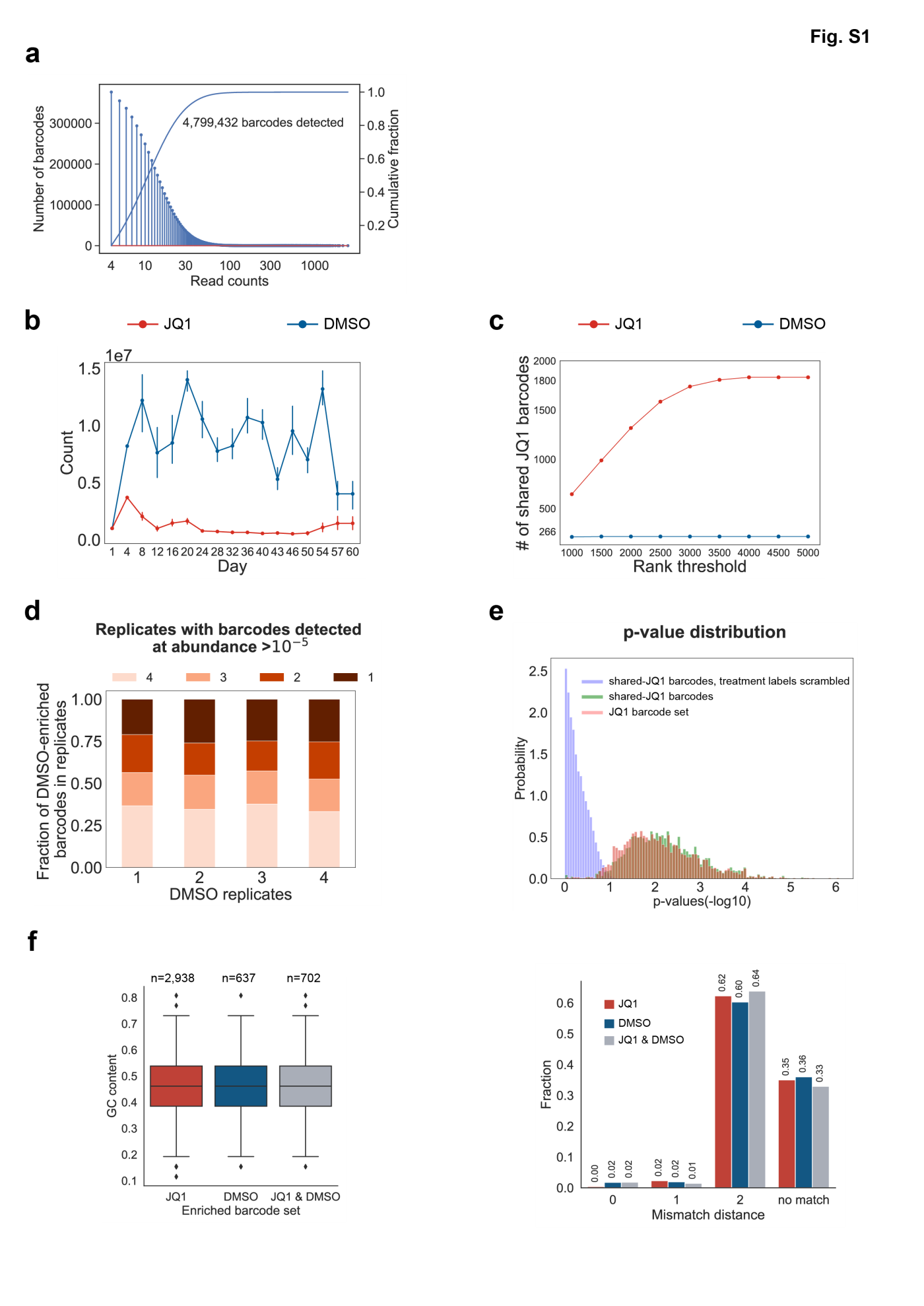
**

#### Fig. S1 sgRNA-barcode library representation, GC bias, and human genome off-targets. (a) Deep sequencing of the 26-nt sgRNA-barcode library plasmid pool. (b) Growth kinetics of D458 cells under DMSO (blue) or JQ1 (red) selection over a period of 60 days. (c) Relationship between median rank threshold and number of JQ1- shared barcodes. Placing the median rank threshold at 2,500 captured the majority of shared-JQ1 barcodes, while increasing the median rank threshold did not substantially increase the number of shared-JQ1 barcodes (red). (d) DMSO-enriched barcodes with abundance higher than 1 in 100,000 were included in the analysis. Less than 30% DMSO-enriched barcodes were shared across 4 replicates. (e) Barcode abundances in the JQ1- and DMSO-treated replicates were compared to generate per-barcode p-values (t-test) indicating the degree of enrichment (green). After scrambling treatment labels, the p-value distribution was shifted to larger values (blue), indicating that barcode enrichments were treatment-dependent. Within the JQ1 barcode set ~90% of barcodes had a *p*-value less than 0.05, consistent with the barcode set definition defined by rank cutoffs (pink). (f) GC content in the 26-nt sgRNA-barcode sequence. There is no significant difference between the barcode sets (p-value = 0.07, one-way ANOVA). Distance of sgRNA-barcodes to the human genome predicted by an off-target sgRNA algorithm. The vast majority of sgRNA-barcodes in the enriched sets have mismatch distance ≥ 2 homology to the human genome.

**
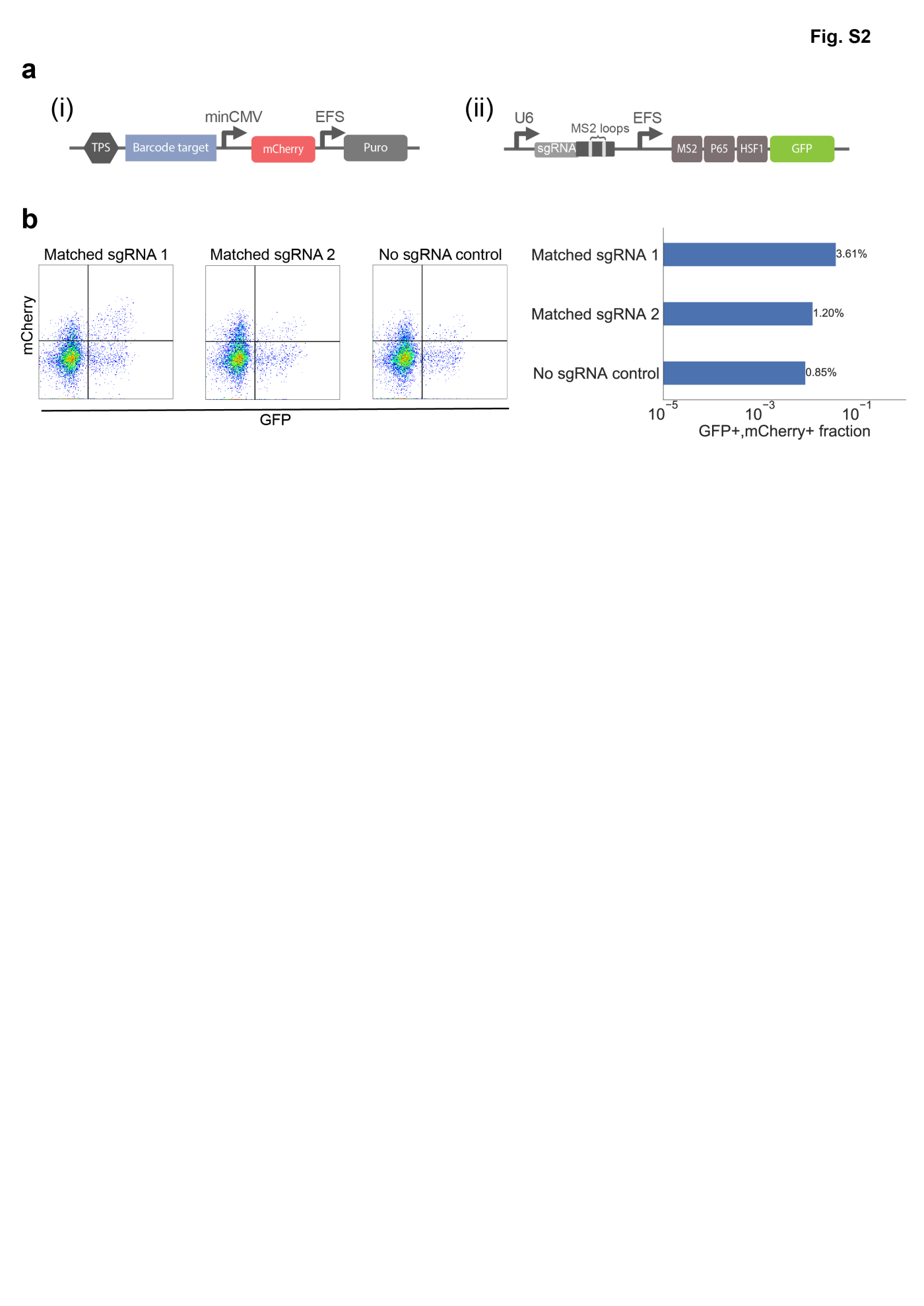
****Fig. S2 Transcriptional activation-based retrieval reporter.** (a) (i) The reporter comprises a transcriptional pause site (TPS), barcode target, minCMV promoter, mCherry fluorescent reporter and puro resistance marker. (ii) sgRNA with transcriptional activator. (b) The reporter selectively induces expression of mCherry in cells with matching sgRNA (Matched sgRNA 1 & Matched sgRNA 2), while cells without the sgRNA sequence exhibit a low level of mCherry expression (No sgRNA control).

####
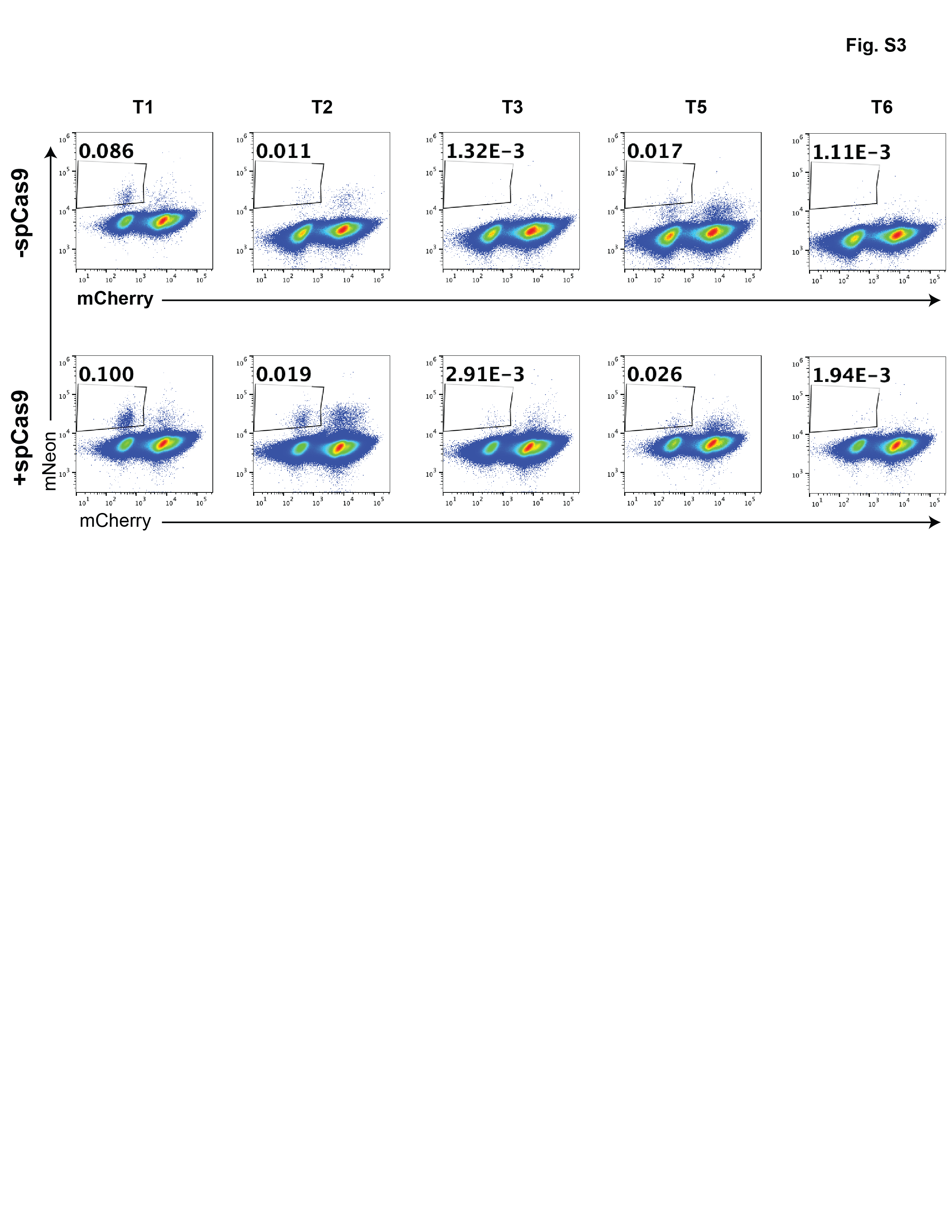


#### Fig. S3 Negative-control groups showed minimal GFP+/mCherry- fraction. Cells were transduced with the retrieval vector containing either matched-barcodes (T1-T4) or barcodes absent in the library (T5-T6). The cells were analyzed for GFP+/mCherry- fraction 9 days after spCas9 induction (lower panel). The cells without spCas9 induction were also analyzed (upper panel). Values are percentages.

####
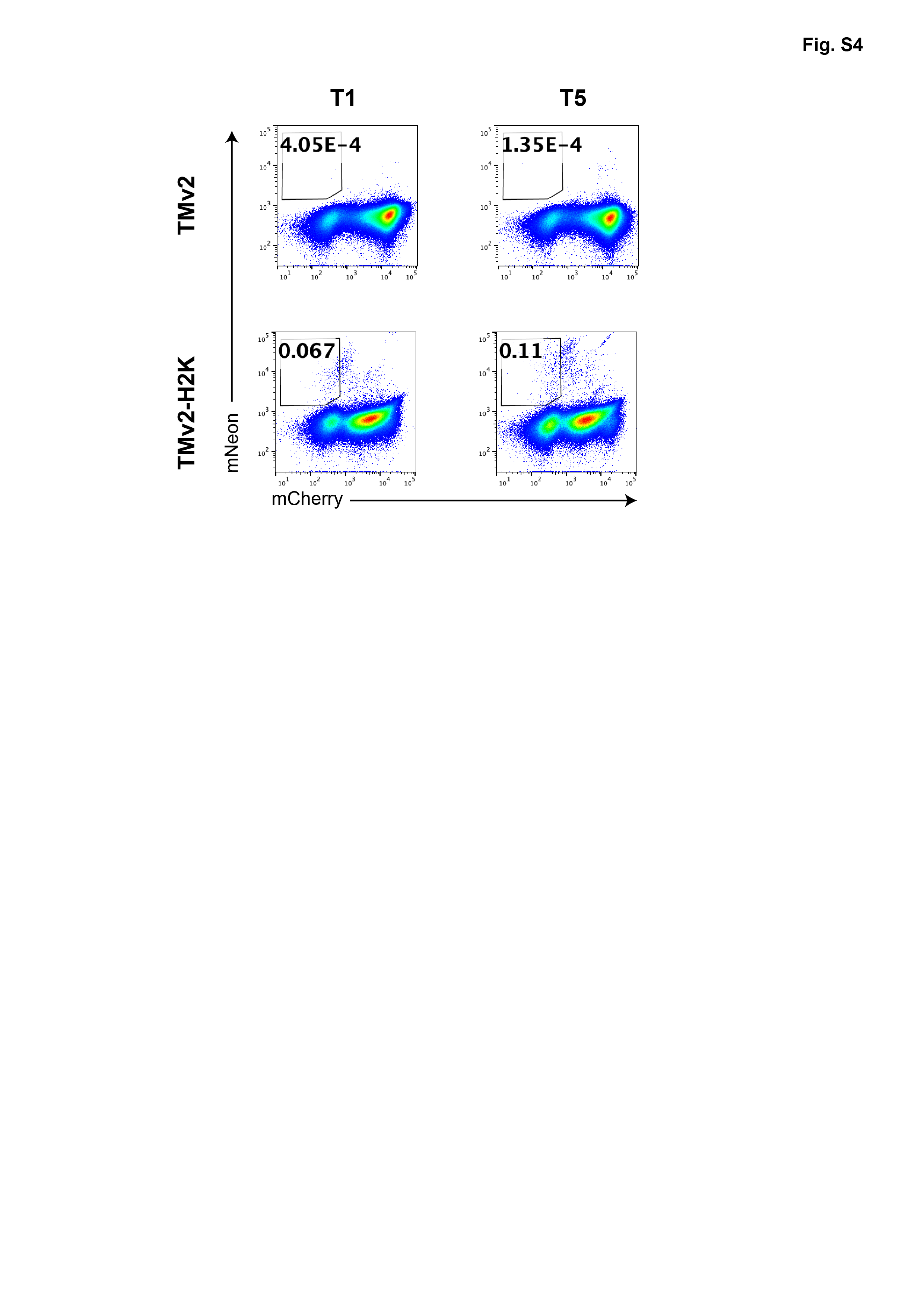


#### Fig. S4 Performance of TMv2-H2K performance in a pooled population. TMv2-H2K was evaluated for specificity with FACS. In the absence of spCas9 TMv2-H2K already showed a high background of GFP compared to TMv2, and thus was not evaluated for retrieval performance. Values are percentages.


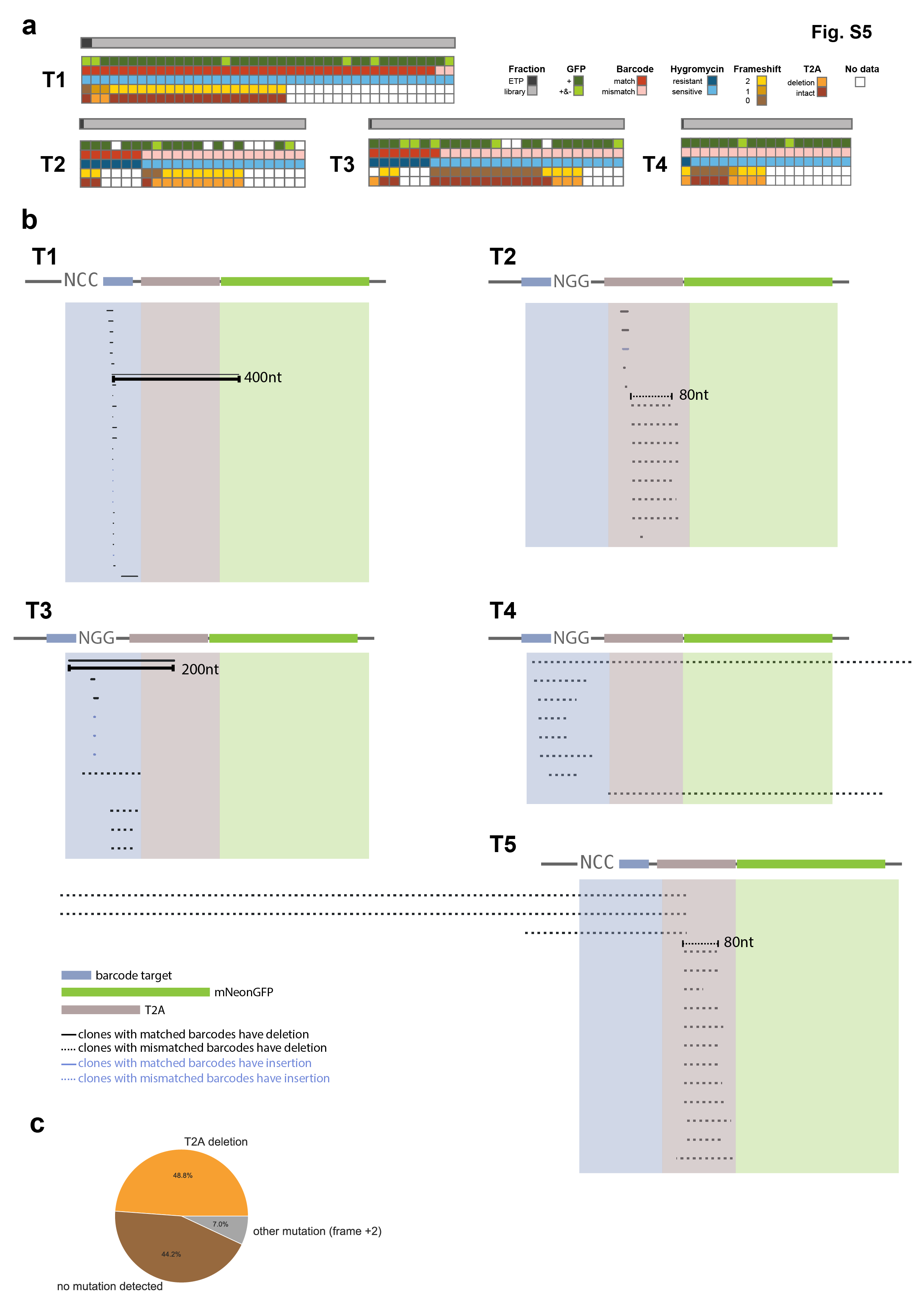


#### Fig. S5 Characterization of retrieved clones. (a and b) Instances of retrieval of clones carrying incorrect barcodes are primarily explained not by FACS error, but by background mutations, in particular a stereotyped T2A linker deletion. (a) Sorted clones were analyzed by FACS for GFP expression (green), validated for barcode accuracy by NGS (red), validated for hygromycin sensitivity (blue) and Sanger sequenced to determine frameshift status (brown) and T2A (orange) deletion. (b) Map of the retrieval vector focusing on the targeting region (blue), T2A (brown), and mNeonGFP (green) for T1-T4 and a non-targeting control (T5). Each line represents a clone, with black and blue lines represent deletion and insertion regions, respectively. Normal and dashed lines represent clones with matched and mismatched barcodes, respectively. The line depicts the location of the deletion or insertion, with the length of the line proportional to the size of the deletion or insertion. Sanger sequencing data for each clone is provided as a SEQ file in Additional file 10: Sanger sequencing files. (c) The types of background in the clones with mismatched barcodes are shown. T2A deletions account for 48.8% of background events.

####
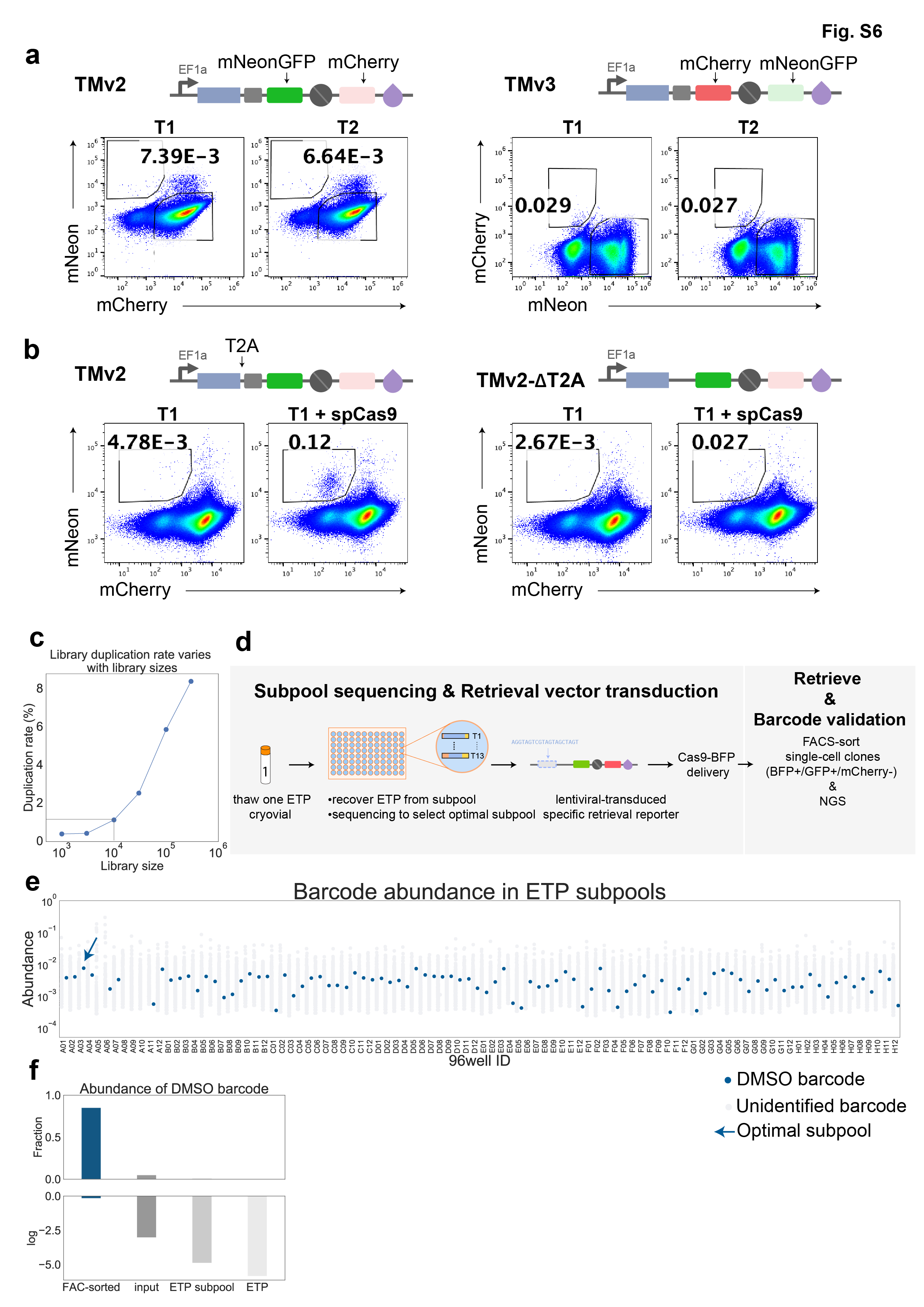


#### Fig. S6 Retrieval vector optimization and retrieval of targeted clones from D458 cells. (a and b) Two alternative designs were evaluated with the goal of increasing specificity by reducing false-positive events measured by FACS. Values are percentages. (a) The alternative design, TMv3 did not show a substantially reduced background in the absence of spCas9, and was not selected for retrieval experiments. (b) The alternative design TMv2-deltaT2A showed reduced background in the absence of spCas9, but failed to efficiently activate the reporter in the presence of spCas9. (c) Library duplication rate increases with the size of the library. The 10^4^-scale library captured the most clonal lineages, as the duplication rate was 1%. (d) Workflow for D458 retrieval. (e) Strip-plot-based representation of barcode abundances in the 96 ETP subpools. The selected-DMSO barcode and other barcodes in each ETP sub-pool. The optimal subpool was indicated by a blue arrow. (f) DMSO barcode was successfully retrieved. The initial abundance of the DMSO barcode in the ETP population was 1 in 346, the abundance in the ETP sub-pool was 1 in 133, and in the input pool was 1 in 20. The retrieval process allowed for enriching the DMSO barcode at >85% purity.

####
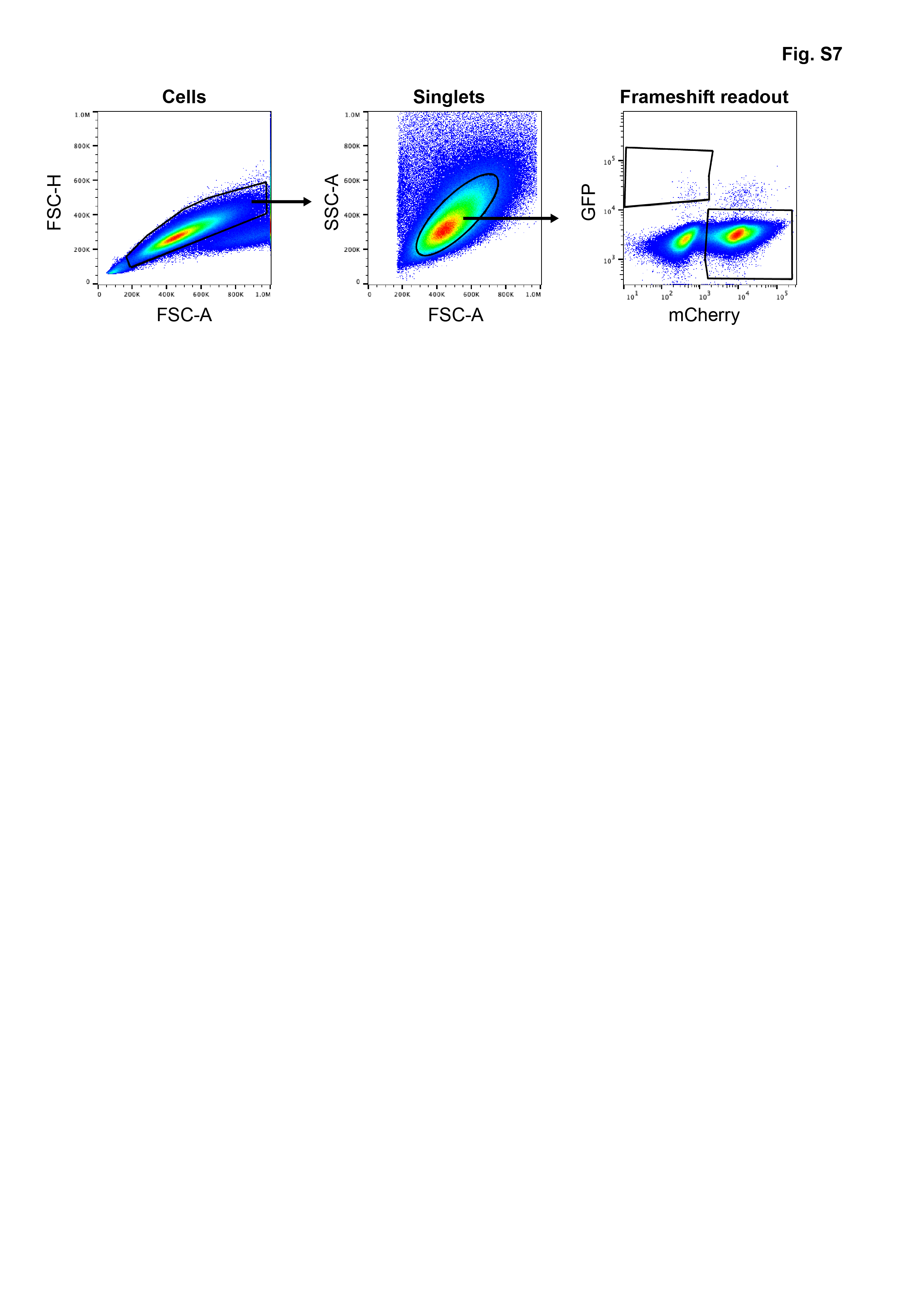
Fig. S7 Gating strategy for analysis of cells with activated frameshift reporter. Representative flow plots for HeLa cells after frameshift mutation-induced with spCas9.

#### Table S1. Table of the clonal barcode candidates, multi-target barcodes and non-targeting barcodes in Figure 3d, Figure 4 and Fig. S3.

| Multiplex target (related to Figure 3d) | | | |
| --- | --- | --- | --- |
| Multi-target | **Barcode** | **sgRNA-barcode** | **Target sequence**  **(PAM lowercase)** |
| MT1 | T319 | GTGTGATCAAATTCGGTGAGGGGGAT | cggatacggacgatcCGTCTCcAAATcgccaccatgctactgtaccc**ccg**ATTCCCTACCCCACTGTCTCcc**ccg**ACTCCCAACTACACGTTTTAcc**ccg**CTTTTCACGACGACCAAACAc**ccg**TTCACCCCTCTCCCTTGGCAcc**ccg**ATCATCTTACCCGTCGACCCcc**ccg**CACTACTTCACTACCTTTTTc**ccg**ATCCCCCTCACCGAATTTGAcctcaagctGTACgGAGACGgatcgtccgtatccg |
|  | T316 | CGTGGTGGGTCGACGGGTAAGATGAT |  |
|  | T318 | GGGTACTAAAACGTGTAGTTGGGAGT |  |
| MT2 | T304 | GGGTTGTTTTGTTCAGCCGTATTGTT | cggatacggacgatcCGTCTCcAAATcgccaccatgctactgtacc**ccg**AAACAGAGCCTAGTCTACATcc**ccg**TAATATTCGTGCCGCATTCGccccgGCCCGCAGCGACACTTACCTcc**ccg**CTAGTCGTAACCACCGACCCcccGTTAATAGAGCGGGAGTTTTcggccTTTTGTTCAGCCGTATTGTT**cgg**c**ccg**ACTATCACATTGAACTCCCActcaagctGTACgGAGACGgatcgtccgtatccg |
|  | T307 | GAGGTGGGGTCGGTGGTTACGACTAG |  |
|  | T308 | GTGGTTGTTAATAGAGCGGGAGTTTT |  |

####

| **Single target (related to Figure 4)** | | | |
| --- | --- | --- | --- |
| **Clonal barcode** | **sgRNA-barcode** | **Target sequence**  **(PAM lowercase)** | **Hygromycin sensitivity** |
| **T1** | AGATCGTACCAGGGATTGGG | GCCACCATGCGCCAGATCGTACCAGGGATTGGG**agg**CTACTGTACCGATCACT | sensitive |
| **T2** | TGCAGTGGCCGTTGACAAAT | GCCACCATGGTACCGCGGG**cct**ATTTGTCAACGGCCACTGCACTACTATCACT | resistant |
| **T3** | GCGGGATCATTGCAATTATA | GCCACCATGGTACCGCGCCGCGGGATCATTGCAATTATA**cgg**CTACTATCACT | resistant |
| **T4** | CAACATCCTGGGGCACAAGC | GCCACCATGCGCCCAACATCCTGGGGCACAAGC**agg**CTACTGTACCGATCACT | resistant |
| **T5** | GATTGGGAGCGTGAGCGTAT | CGCCACCATGCGA**ccg**ATCGGCCTATACGCTCACGCTCCCAATCCTACTGCACT | non-targeting control |
| **T6** | GGGACGCACTGCAGAGTGTA | CGCCACCATGCGCCGGGACGCACTGCAGAGTGTA**cgg**CTACTGTACCGATCACT | non-targeting control |
| **T7** | AGGGGGCTGACGGGGCGTTA | n/a | resistant |
| **T8** | CAAAGCCAGTGACGATAATT | n/a | resistant |
| **T9** | TTCATTCAGCGCCATGCCCC | n/a | resistant |
| **Multiplex target (related to Figure 4)** | | | |
| **4-multiplex**  **T3, T7, T8, T9** | N/A | cgccaccatgctGCGGGATCATTGCAATTATA**cgg**cTTCATTCAGCGCCATGCCCC**cgg**cCAAAGCCAGTGACGATAATT**cgg**cGGGGGCTGACGGGGCGTTA**cgg**cAGGGGGCTGACGGGGCGTTA**cgg**cactgtaccgatcaagcttc | N/A |
| **2-multiplex**  **T3, T7** | N/A | cgccaccatgctGGGGGCTGACGGGGCGTTA**cgg**cGCGGGATCATTGCAATTATA**cgg**cactgtaccgatcaagcttc | N/A |

#### Table S2. Enrichment levels of barcodes that are retrieved with a 4-multiplex TMv2-Zeo or a 2-multiplex TMv2-Zeo.

| Reporter vector | **Clonal barcode** | **Input abundance** | **Output abundance** | **Fold enrichment** |
| --- | --- | --- | --- | --- |
| 4-multiplex TMv2-Zeo | T3 | 0.00023966 | N/A | N/A |
|  | T7 | 0.00312095 | 0.46494215 | 148.974329 |
|  | T8 | 0.00126653 | 0.00173631 | 1.37091676 |
|  | T9 | 0.00016598 | 0.00031342 | 1.88835373 |
| 2-multiplex TMv2-Zeo | T3 | 0.00019161 | 0.00843461 | 44.0205391 |
|  | T7 | 0.0037865 | 0.93369485 | 246.584985 |

####

#### Table S3. Table of primer sequences used for amplifying 2 kb-lentiviral transgene and for Sanger sequencing.

| Primers | **Sequence (5’- 3’)** |
| --- | --- |
| pTM_negative_fwd | TCTTTCCCTACACGACGCTCTTCCGATCTAGCAGAGATCCAGTTTGGTTAATTAGCTAGC |
| pTM_negative_rev | AAGACTACAGCGTCGCCAGCAGATCGGAAGAGCACACGTCTGAACTCCA |
| pTM_sanger_primer | GGATCTTGGTTCATTCTCAAGCC |

#### 
